# Lipid Dependence of CYP3A4 Activity in Nanodiscs

**DOI:** 10.3390/biology15020156

**Published:** 2026-01-15

**Authors:** Ilia G. Denisov, Yelena V. Grinkova, Stephen G. Sligar

**Affiliations:** 1 Department of Biochemistry, University of Illinois, 116A Morrill Hall, 505 S. Goodwin Ave., Urbana, IL 61801, USA; denisov@illinois.edu (I.G.D.);; 2 Department of Chemistry, University of Illinois, 505 S. Mathews Ave., Urbana, IL 61801, USA

**Keywords:** P450 CYP3A4, Nanodiscs, lipid effects on metabolism, allosteric site, steroid hydroxylation, midazolam regiospecificity

## Abstract

All human cytochrome P450 enzymes are incorporated into a membrane with important functional implications. In some P450 enzymes, interactions of the F-G loops with lipid head groups create a peripheral allosteric site at this protein–lipid interface. In addition, the redox potential of CYP3A4 and cytochrome P450 reductase (CPR) is modulated by negatively charged lipids in the membrane. In order to investigate the effect of lipid headgroups on human CYP3A4 activity, we studied the rate of NADPH oxidation and product formation using progesterone and midazolam as substrates, assembled into Nanodiscs with different lipid compositions. Preparation of a polar lipid extract provided a modest acceleration of both NADPH oxidation and the rate of progesterone hydroxylation, compared to the zwitterionic palmitoyl-oleoyl phosphatidyl choline (POPC). The highest activity was found in Nanodiscs assembled with liver polar extract lipids together with negatively charged phosphatidylserine (PS). No effect of lipid composition was detected for the regiospecific 1OH/4OH hydroxylation ratio of midazolam (MDZ) metabolites, indicating the absence of membrane composition perturbation of the allosteric effects in MDZ hydroxylation. The magnitude of the observed effect of lipid composition on the catalytic rate of CYP3A4 strongly depends on the substrate used as a probe.

## Abstract (plain language summary)

About a dozen cytochrome P450 enzymes metabolize more than a half of all pharmaceuticals in the human liver. The most important of these enzymes is CYP3A4, which is involved in removal of hundreds of drugs and other chemical compounds from the human body. Cytochrome P450 enzymes are connected to the lipid membranes and various lipid head groups affect their functional properties. We used CYP3A4 incorporated in the soluble membrane Nanodiscs with controlled lipid compositions to probe the effect of various lipids on activity of this enzyme. Our results show that the effect depends on the structure of substrate used for the functional studies. Very small changes were detected with the specific substrate midazolam, which is used as a probe in clinical practice. However, a significant dependence of activity of CYP3A4 on the lipid composition was observed with progesterone as a substrate, because of modulation of the enzyme functional properties by the binding of this molecule at the protein–membrane interface. These novel results shed light on the important role of CYP3A4 interactions with the lipid membrane in drug metabolism studies.

## 1. Introduction

CYP3A4 is the main xenobiotic metabolizing enzyme in the human body, as it is in volved in metabolism of ~30% of drugs on the market as well as of many other compounds, from small molecules such as ethanol to large drugs such as erythromycin and cyclosporine [1,2,3,4]. As all other mammalian cytochrome P450 enzymes, CYP3A4 is a peripheral membrane protein, mostly found in the endoplasmic reticulum in the liver [5]. Because of its large and flexible active site, CYP3A4 can, and often does, bind more than one substrate molecule, resulting in a versatile pattern of homotropic and heterotropic cooperative effects observed in functional studies, both in vitro, as well as in vivo [6,7,8,9,10,11,12,13,14,15,16,17].

The activity of cytochrome P450 enzymes can be studied in human liver microsomes, hepatocytes, and baculosomes. However, these systems contain a plethora of other biomolecules, and detailed control of the local environment of cytochrome P450 and redox partners, as well as the stoichiometry of protein–protein complexes, is difficult. Biophysical and biochemical studies of purified mammalian cytochrome P450 enzymes often use various detergent-solubilized systems, often with the addition of lipids, although in such systems, the membrane lipid bilayer is not present or significantly perturbed. The Nanodisc approach provides a well-characterized patch of lipid bilayer with known composition. Monomeric cytochrome P450, co-incorporated with its redox partner protein P450 reductase, forms stable functional complexes for a wide variety of biochemical and biophysical studies [9,18,19]. An important advantage of the Nanodisc system is its ability to control lipid composition for illuminating the effect of the membrane on CYP3A4 activity and allosteric properties.

Membrane properties and lipid composition play an important role in functional properties of cytochrome P450 enzymes and their redox partners. The interactions of cytochrome P450 enzymes with the membrane change their dynamics and stability [20,21]. Many hydrophobic substrates which are poorly soluble in water are preferentially partitioned into the membrane bilayer and likely bind to the active site of P450 enzymes via a substrate access channel directed towards the membrane [22,23,24,25]. In addition, CYP3A4, and possibly some other cytochrome P450 enzymes, have an external allosteric site between the F-G helices and the lipid head groups. Interactions of cytochrome P450 and P450 reductase can be modulated by the membrane lipids, as shown experimentally for both CYP2B4 and CYP3A4 [26,27,28]. These protein–membrane interactions depend on the lipid composition of the membrane bilayer and can significantly affect the activity and allosteric properties of cytochrome P450 enzymes, which are also dependent on the substrate being metabolized. For instance, substrate properties, such as hydrophobicity, preferential partitioning into the lipid bilayer, and specific binding sites on a given P450 enzyme, can be critical.

In this publication, we use the Nanodisc system for a systematic study of the effect of the membrane lipid composition on the activity and allosteric properties of CYP3A4. Previously, we demonstrated that CYP3A4 can bind up to three molecules of a steroid substrate, such as testosterone or progesterone (PROG) [9,29,30,31,32], or two molecules of midazolam (MDZ) [13,14]. MDZ can be used as a specific substrate probe to monitor CYP3A4 activity *in vivo* where we demonstrated that the ratio of two products of MDZ hydroxylation at the C1 and C4 positions, the 1OH/4OH ratio, can be used as a sensitive probe for the allosteric interactions of CYP3A4 with other drugs and xenobiotics [33]. In order to investigate the possible effect of the lipid composition of the membrane on the sensitivity and reproducibility of the MDZ-based drug interaction probe, we performed a series of functional studies of CYP3A4 in Nanodiscs assembled with various lipids. We used a 100% zwitterionic palmitoyl-phosphatidylcholine (POPC) as a reference, and studied the effect of mixtures of 40% dioleoyl-phosphatidylethanolamine (DOPE) and 40% *dioleoyl*-phosphatidylserine (DOPS) with 60% POPC. In addition, we include Nanodiscs assembled using a bovine liver polar lipid extract, to provide a closer model for the native membrane environment of CYP3A4.

## 2. Methods

### 2.1. Protein Expression and Purification

Expression and purification of the membrane scaffold protein (MSP) used for Nanodisc formation, cytochrome P450 CYP3A4 and rat P450 reductase, as well as assembly of CYP3A4 into Nanodiscs (ND) was executed following previously described protocols [33,34]. Briefly, cytochrome P450 CYP3A4 was expressed from the NF-14 construct in the PCWori+ vector with a C-terminal pentahistidine tag generously provided by Dr. F. P. Guengerich (Vanderbilt University, Nashville, TN, USA). Monomeric CYP3A4 in Nanodiscs with a diameter of 10 nm was isolated using an affinity column followed by size exclusion chromatography as described in [33,34]. Cytochrome P450 reductase (CPR) was expressed using the rat CPR/pOR262 plasmid, a generous gift from Dr. Todd D. Porter (University of Kentucky, Lexington, KY, USA). Incorporation of CPR into preformed and purified CYP3A4-Nanodiscs was made by direct addition of CPR at a 1:4 CYP3A4/CPR molar ratio, as described in [19]. All experiments were performed at 37 °C using Nanodiscs analogous to our earlier detailed mechanistic studies [9,30]. POPC, DOPE, and DOPS lipids were purchased from Avanti (Alabaster, AL, USA). Bovine liver polar lipid extract from Avanti (lot #181108) contained 42% PC, 26% PE, 9% PI, 1% lyso PI, 5% cholesterol, and 17% other neutral lipids (information from the Supplier site https://avantiresearch.com/product/181108, accessed on 21 November 2025). Concentrations of all lipids were measured using the phosphate analysis method as described in [9]. Concentrations of substrate-free CYP3A4 in ND were measured by absorption at 417 nm using a molar extinction coefficient of 110 mM^−1^cm^−1^ (Figure S1).

### 2.2. NADPH Oxidation and Product Formation

CYP3A4-incorporated Nanodiscs with CPR added at a 1:4 molar ratio, in the presence of substrate, were preincubated for 5 min at 37 °C, in a 1 mL reaction volume in 100 mM HEPES buffer (pH 7.4) containing 10 mM MgCl_2_ and 0.1 mM dithiothreitol. The concentration of CYP3A4 was in the range from 60 to 100 nM. The reaction was initiated with the addition of 200 nmol of NADPH. NADPH consumption was monitored for 5 min and calculated from the absorption changes at 340 nm using an extinction coefficient of 6.22 mM^−1^ cm^−1^.

For product analysis, the reactions were run in volumes of 80–100 μL in microcentrifuge tubes. At the end of the incubation period, the reactions were quenched by addition of 20 μL of acetonitrile containing internal standard (37 μM nordiazepam). The samples were centrifuged at 3000× *g* for 30 min, and 20–40 μL of supernatants was injected into the Ace 3 C18 HPLC column, 2.1 × 150 mm (MAC-MOD Analytical, Chadds Ford, PA, USA), on an LC-20LD chromatograph (Shimadzu, Columbia, MD, USA). The mobile phase contained 22% acetonitrile and 28% methanol in water; products of PROG hydroxylation and MDZ hydroxylation were separated at a flow rate of 0.15 mL/min as follows: isocratic separation for 20 min, and then linear gradient for 5 min where concentrations of acetonitrile and methanol were raised to 50% and 32%, respectively, followed by second isocratic separation for 12 min. The calibration and method validation were performed using commercially available metabolites of PROG and MDZ. The chromatograms were processed with Shimadzu software LabSolutions, v. 5.87. The standard deviations of several independent measurements ranged from 10% to 20% of the calculated rates of product formation.

### 2.3. Global Analysis for Deconvoluting Apparent Cooperative Effects in P450

Global analysis of homotropic cooperativity for the metabolism of MDZ was performed, as previously described [9], by simultaneously fitting the experimental data sets to the three-state linear equilibrium binding scheme:

E + S → ES + S → ES_2_

K_1_    K_2_

where E is the concentration of substrate-free CYP3A4; S is the concentration of the free substrate; ES_i_ are the concentrations of the binding intermediates, i.e., complexes of CYP3A4 with i molecules of substrate bound (i = 1, 2); and K_1_ and K_2_ are dissociation constants for the first and second binding steps. The binding of up to three molecules of substrates to one CYP3A4 monomer was described in previous studies [9,31,32], although for the case of MDZ homotropic cooperativity described in this communication, only two are required. The fractions of the enzyme–substrate complexes are expressed using the standard binding polynomials [9,11]:

$$Y=\frac{\frac{S}{K_{1}}+\frac{S^{2}}{K_{1}K_{2}}}{1+\frac{S}{K_{1}}+\frac{S^{2}}{K_{1}K_{2}}}$$

with the functional properties at different substrate concentrations represented as the linear combination of the fractional contributions from binding intermediates, where K_i_ are stoichiometric dissociation constants. For example, the rate of NADPH oxidation by CYP3A4, V_N_, as a function of substrate concentration S is calculated as the weighted sum of the fractional contributions from the cytochrome P450 molecules with 0, 1, or 2 substrate molecules bound, with v_0_, v_1_, and v_2_ rates:

$$V_{N}=\frac{v_{0}+\frac{v_{1}S}{K_{1}}+\frac{v_{2}S^{2}}{K_{1}K_{2}}}{1+\frac{S}{K_{1}}+\frac{S^{2}}{K_{1}K_{2}}}$$

The set of such equations for spectral titration, NADPH consumption, and product formation were used for the simultaneous fitting of the experimental data obtained under the same conditions, and hence with the same set of dissociation constants. All experimental results used in fitting were normalized to the same scale in order to avoid the dominant contribution of large numbers to the total mean square deviation [9]. The fitting program was written in MATLAB version R2021b using the Nelder–Mead simplex minimization algorithm implemented in the MATLAB subroutine “fminsearch.m.”

Analysis of PROG metabolism was performed using a similar approach for three-site binding, as described before [9,30,31]. In such cases, the equilibrium substrate binding is described by four states with zero, one, two, or three substrates bound to the CYP3A4 molecules:

E + S → ES + S → ES_2_ + S → ES_3_

K_1_    K_2_   K_3_

The experimentally observed rates are calculated as a sum of the fractional contributions from all binding intermediates:

$$V_{N}=\frac{v_{0}+\frac{v_{1}S}{K_{1}}+\frac{v_{2}S^{2}}{K_{1}K_{2}}+\frac{v_{3}S^{3}}{K_{1}K_{2}K_{3}}}{1+\frac{S}{K_{1}}+\frac{S^{2}}{K_{1}K_{2}}+\frac{S^{3}}{K_{1}K_{2}K_{3}}}$$

## 3. Results

**Figure 1:**
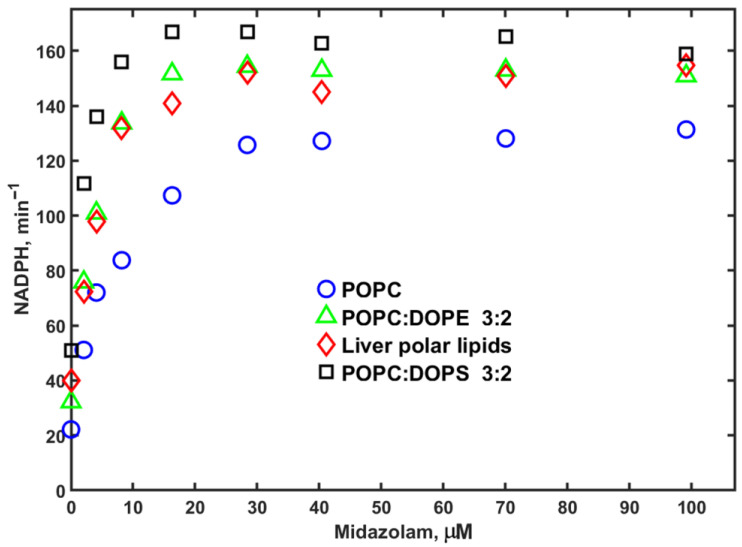
NADPH oxidation rates as a function of substrate concentration as measured for MDZ hydroxylation by CYP3A4 in Nanodiscs with various lipid compositions and CYP3A4:CPR ratio 1:4.

**Figure 2:**
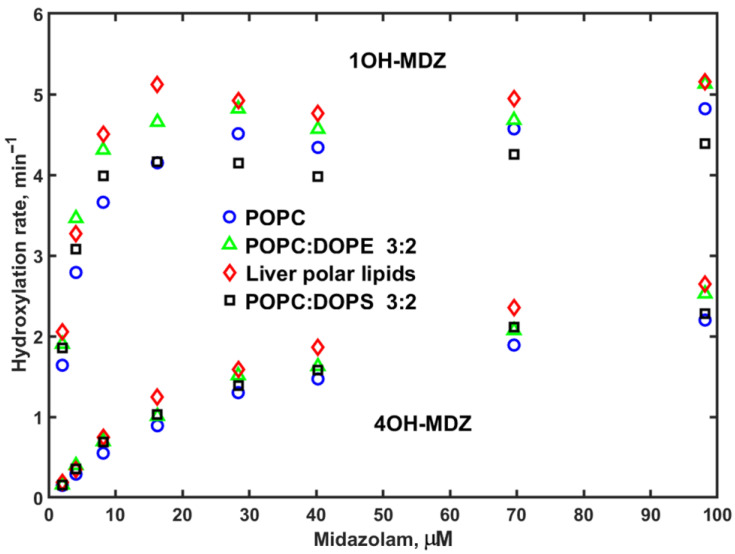
Product forming rates as a function of MDZ concentration measured for 1OH hydroxylation and 4OH hydroxylation by CYP3A4 in Nanodiscs with various lipid compositions and CYP3A4:CPR ratio of 1:4.

In order to explore the effect of membrane lipid composition on the activity of CYP3A4, we measured the rates of MDZ and PROG hydroxylation by CYP3A4 co-incorporated with cytochrome P450 reductase (CPR) in Nanodiscs with various lipid compositions as a function of substrate concentration. We included 100% POPC as a reference system as used in our previous studies. In addition, we assembled Nanodiscs with mixtures of 40% DOPE or 40% DOPS with 60% POPC, and also prepared Nanodiscs using a polar lipid extract from bovine liver (Avanti, #181108). The results obtained with MDZ as a substrate are shown in Figure 1 and Figure 2. The experimentally observed rates of NADPH consumption were found to be lowest in POPC Nanodiscs and highest when 40% DOPS was added. Overall, however, the differences in these rates are not remarkably large, although they are consistently observed at all substrate concentrations.

The product forming rates of MDZ hydroxylation, as a function of substrate concentration, are shown in Figure 2. The lipid composition effect on these rates is also not large, but different from the NADPH consumption rates. Both 1OH and 4OH hydroxylated midazolam products are formed slightly faster in Nanodiscs assembled using the liver polar lipids (LPLs), while the activity of CYP3A4 in 40% DOPS is slightly lower. Overall, comparison of these two data sets indicates no large effect of lipid composition on the absolute rates of MDZ metabolism.

**Figure 3:**
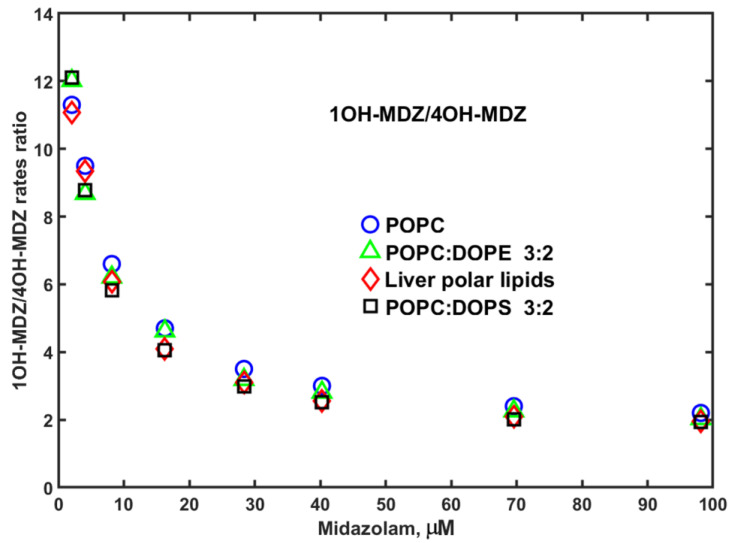
1-OH/4-OH product forming rate ratio vs. MDZ concentration measured for hydroxylation of MDZ catalyzed by CYP3A4 in Nanodiscs with various lipid compositions and CYP3A4:CPR ratio of 1:4.

Since no significant differences in metabolism rates were detected in Nanodiscs assembled with various lipids, the same absence of a large effect of lipid composition is noted on the metabolic ratio, i.e., the ratio of MDZ hydroxylation rates at C1 and C4 positions, 1OH/4OH, by CYP3A4 in Nanodiscs (Figure 3).

The higher rates of MDZ hydroxylation in Nanodiscs assembled with LPL may be attributed to the better interactions of CYP3A4 with CPR, also seen in PROG hydroxylation experiments (see below). The regioselectivity measured as the ratio of hydroxylation rates at C1 and C4 positions remains almost the same for all lipid compositions, supporting the validity of MDZ to be used as a probe for drug–drug allosteric interactions, as described in [14].

**Scheme 1:**
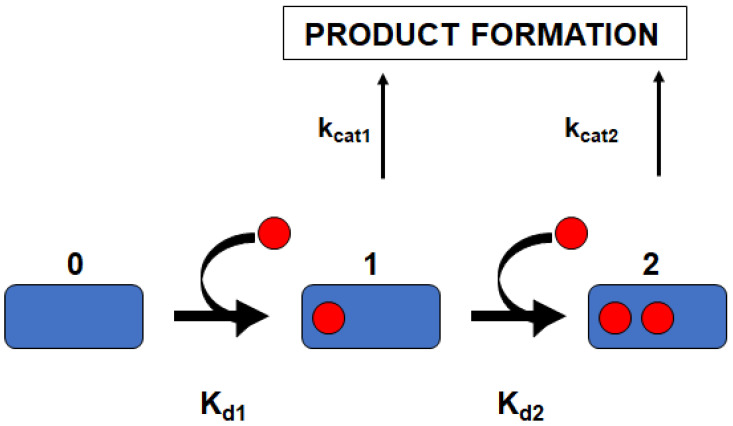
Stepwise binding of two-substrate molecule to the CYP3A4 monomer with corresponding dissociation constants K_d1_ and K_d2_ and fractional product forming rates k_cat1_ and k_cat2_ by CYP3A4 with one or two substrates bound. The total experimentally observed rate of product formation is equal to k_cat1_ + k_cat2_. Hydroxylation rates at C1 and C4 positions and NADPH oxidation rates as functions of substrate concentrations are fitted simultaneously using parameters shown in Table 1 (see Section 2 for the details).

In order to obtain more detailed quantitative measures of the lipid effect on the parameters of MDZ hydroxylation by CYP3A4, we performed a simultaneous fitting of the NADPH oxidation and product formation rates using a two-site model, Scheme 1, as described in Section 2 and in our earlier works [13,14].

The results of the global fitting of MDZ hydroxylation experimental data are shown in Table 1 and the figures in the Supplemental Information (Figures S2–S5). Overall, the lipid composition does not seem to play an important role in MDZ binding and the rates of NADPH oxidation in hydroxylation of midazolam. The binding of the first MDZ molecules is a little tighter in Nanodiscs with 40% DOPS (K_d1_ = 1.8 μM), and the second MDZ molecule binds a little tighter in LPL Nanodiscs (K_d2_ = 21.5 μM), as compared to binding in POPC and 40% DOPE Nanodiscs. The coupling of NADPH oxidation with product forming rates calculated as the ratios of product forming rates to the NADPH consumption rates is low, ranging from 2.2% to 5.4%; the second MDZ binding slightly improves overall coupling, which was also observed in several other cases when the fractional contributions to the overall product forming rates were resolved [9,12,30,31].

**Figure 4:**
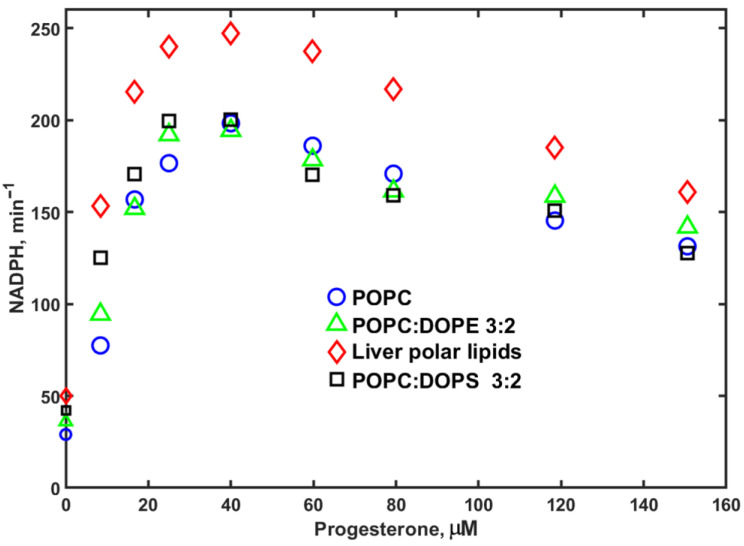
NADPH consumption rates as a function of PROG concentration measured for PROG hydroxylation catalyzed by CYP3A4 in Nanodiscs with various lipid compositions and CYP3A4:CPR ratio of 1:4.

The same measurements of NADPH oxidation and hydroxylation rates by CYP3A4 in Nanodiscs with various lipid compositions were performed using PROG as a substrate (Figure 4).

Samples with Nanodiscs assembled using liver polar lipids (LPLs) showed slightly higher maximal rates at all lipid concentrations, while other lipids were roughly the same as the POPC control. However, at low substrate concentrations, there is a significant difference, with the NADPH consumption rate two times lower in POPC than in LPL, while in DOPS, the rates are significantly faster. Similar results are observed for NADPH consumption rates with MDZ as a substrate, although the lipid-dependent difference is less pronounced.

**Figure 5:**
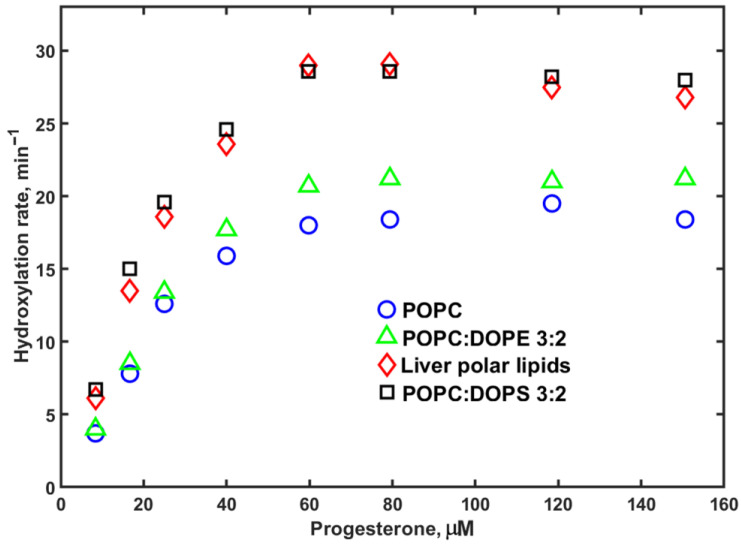
Rates of PROG hydroxylation catalyzed by CYP3A4 in Nanodiscs with various lipid compositions and CYP3A4:CPR ratio of 1:4, as a function of PROG concentration.

The rates of PROG hydroxylation by CYP3A4 in Nanodiscs assembled using LPL and 40% DOPS are also significantly higher than those measured in Nanodiscs with 40% DOPE or 100% POPC. This difference is observed at all substrate concentrations, but is most pronounced at low and intermediate PROG concentrations, as seen in Figure 5.

Overall, the effect of lipid composition on CYP3A4-catalyzed hydroxylation reactions is more pronounced when PROG is used as a substrate, than for MDZ hydroxylation.

More specific information about the lipid effect on the parameters of CYP3A4 catalyzed hydroxylation of PROG was obtained using a global analysis of all experimental data shown in Figure 4 and Figure 5 using a three-site model, as described in Section 2 and in our earlier work [9,29,31]. The results of this analysis are summarized in Table 2 and Figures S6–S9. NADPH oxidation rates were divided by 5, as shown in Figures S6–S9, in order to adjust their contribution to the calculated mean square deviation in the global fitting procedure, as described earlier [9,31].

The results from global fitting for CYP3A4 in Nanodiscs assembled using the liver polar lipid extract show a significant difference both in the stepwise substrate binding constants and in the rates of NADPH consumption and PROG hydroxylation. The affinities of the second and third substrate binding steps are lower than those observed with other lipid compositions (Table 2), while the rates of hydroxylation are higher than in POPC and in DOPE Nanodiscs. An exception is the intermediate with three substrates bound, where the rate v_3_ is approximately two times lower. Hence, the coupling ratios are also significantly higher in LPL Nanodiscs, similarly to the values measured in 40% DOPS Nanodiscs.

We showed earlier that in Nanodiscs assembled with POPC, the binding of steroid substrates TST and PROG first occurs at the high-affinity peripheral site between F’ and G’ helices and lipid head-groups [9,31,32]. This site is far from the heme iron and does not generate any product, as shown in Table 2 for the POPC sample, k_cat1_ = 0. The results obtained with 40% DOPE Nanodiscs also show that the first binding event does not result in any product formation. However, CYP3A4 in LPL and in 40% DOPS Nanodiscs demonstrates substantial hydroxylation of PROG even when only one substrate molecule is bound, with k_cat1_ values of 10.3 and 16.9 min^−1^. The significant difference in CYP3A4 functional properties induced by different membrane lipid compositions is an important new result of this study discussed in more detail below.

## 4. Discussion

The importance of lipid interactions in the functional properties of membrane proteins and multiple aspects of protein–lipid interactions constitute a broad area of biophysical and biochemical studies, with many books and review articles covering various topics, from structure and dynamics to functional studies [35,36,37,38,39,40,41,42,43]. Nanodiscs became a valuable tool for the detailed characterization of membrane proteins and their interactions with lipids on a molecular level, as extensively reviewed in [44,45,46,47,48]. Examples include interactions and protein complexes of the cancer-related small GTPase Kras, mediated by specific anionic lipids [49,50,51], the dynamics and allosteric regulations of G-protein-coupled receptors [52,53,54], and the initiation of transmembrane transport and lipid-mediated signaling by scramblases and flippases [55,56].

The effect of membrane lipid composition upon the activity of human cytochrome P450 enzymes is an important factor in mechanistic studies of these enzymes. For example, the critical interactions of P450 heme enzyme with redox partners CPR and cytochrome b5, as well as the binding of hydrophobic substrates, which are preferentially partitioned into the membrane, can be significantly modulated by the lipid environment. The charge and structure of the lipid head-groups and their mobility, as well as interactions of the transmembrane helices of P450 and CPR inside the lipid bilayer, are important. The redox potentials of P450 hemes, and flavin cofactors of CPR, have been shown to be shifted by the electrostatic field of the charged bilayer [57,58], which may significantly accelerate the first and second electron transfer steps of the P450 cycle. Such enhancement in the electron transfer from CPR to CYP3A4 co-incorporated in Nanodiscs with liver polar lipids was also reported by Scrutton and colleagues [28], together with the acceleration of metabolism of a model fluorescent substrate. Interestingly, these authors did not observe such acceleration in Nanodiscs assembled using the model lipid mixture mimicking the natural lipids extracted from bovine liver microsomes. However, this model lipid mixture did not contain cholesterol, which may be an important effector for P450 activity in membranes, as reported for CYP19A1 [59]. In this study, the 3-fold acceleration of the first electron transfer from CPR to CYP19A1 was observed in the presence of cholesterol in the Nanodisc bilayer and is attributed to the increased membrane dipole potential due to the lipid ordering caused by cholesterol, or to the tighter protein–protein interactions.

Another example is the modulation of catalytic properties of the human cytochrome P450 CYP2J2 by negatively charged POPS and by cholesterol in Nanodiscs using ebastin as a substrate [60]. In this system, significant acceleration of ebastin hydroxylation was observed in the presence of 20% and 40% of POPS, while the effect of cholesterol up to 20% was less pronounced.

**Figure 6:**
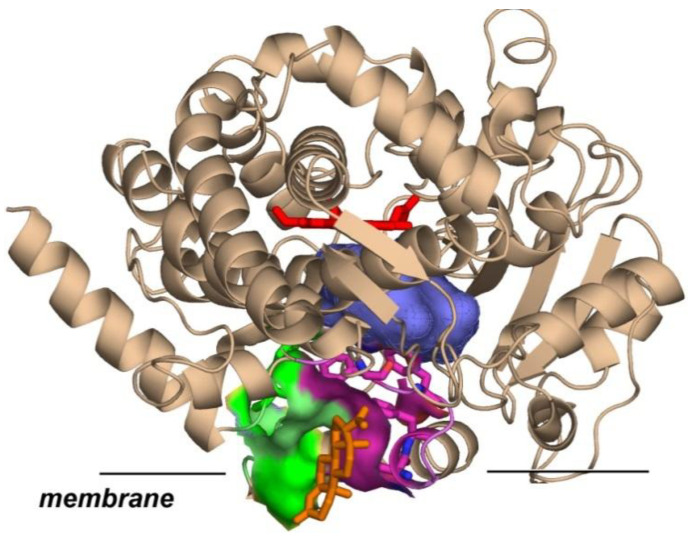
Schematic model of cytochrome P450 CYP3A4 inserted in the membrane (reproduced from [12] with permission). CYP3A4 structure with progesterone (orange sticks) bound at the peripheral site (1W0F), with heme shown in red sticks. The membrane insertion is schematically depicted according to molecular dynamics simulations [31]. Amino acids of F-F’ (magenta) and G-G’ (green) loops in direct contact with progesterone are highlighted as surfaces. The substrate binding cavity near the catalytic site is shown as a blue surface.

In this communication, we provide evidence for a strong dependence of lipid composition on the functional properties of CYP3A4. The rates of NADPH consumption are affected in a similar way for MDZ and PROG in Nanodiscs with different lipids, indicating a modulation due to anionic lipids on the CYP3A4–CPR functional interactions. However, the acceleration of the product formation in DOPS and LPL Nanodiscs is much more pronounced with PROG as a substrate, as compared to MDZ. This difference is likely due to the ability of PROG and other steroids to bind with high affinity at the peripheral allosteric site formed by the F’ and G’ helices of CYP3A4 and lipid head-groups [31,32] (Figure 6).

Substrate binding at the peripheral site, far from the heme, is not productive, and the earlier results of global analysis obtained using POPC Nanodiscs confirm that no product is formed by CYP3A4 with one steroid substrate bound [9,31,32]. When 40% DOPS or LPL is used, some product is generated already from CYP3A4 with one PROG molecule, as shown in Table 2. The NADPH consumption rates in these samples are also significantly higher. These results indicate the redistribution of substrate from the allosteric peripheral non-productive site to the productive position near the heme, due to different lipid head-groups and therefore the lower affinity of this peripheral site to the steroid substrate. The same difference is observed for the product forming rates from the fraction of CYP3A4 with two PROG molecules bound, with much faster hydroxylation rates measured in the samples where LPL or 40% DOPS was used for Nanodisc assembly.

The effect of the lipid head-groups on the activity of CYP3A4 with MDZ as a substrate is much less pronounced. Variations in NADPH consumption rates by CYP3A4 in Nanodiscs with various lipid compositions are less than two-fold. The rates of MDZ hydroxylation are also not significantly different for both fractions of CYP3A4 with one and two substrates bound (Table 1). This small difference, and the lack of a significant effect of the lipid head-groups on CYP3A4-catalyzed hydroxylation of MDZ, unlike in the case of PROG, can be attributed to the difference in affinity of the allosteric peripheral site for MDZ and PROG. For PROG, and other steroids, the binding to this allosteric non-productive site occurs with high affinity when POPC is used for CYP3A4 incorporation in Nanodiscs [30,31,32,34]. Variations in the size and charge of lipid head-groups can change the affinity of this site for PROG and therefore modulate the fraction of productive binding of this substrate to the active site and facilitate product formation even at low PROG concentrations, as observed for 40% DOPS and for LPL-incorporated CYP3A4 (Table 2). At higher substrate concentrations, with the increase in population of the fraction of CYP3A4 with two substrate molecules bound, a difference in hydroxylation rates is also evident, with much faster product turnover rates and concomitant higher coupling ratios in 40% DOPS and in LPL Nanodiscs. Slower product turnover rates observed at the substrate saturation from CYP3A4 with three PROG molecules bound can be attributed to the limitations of the product release pathways caused by the presence of the substrate molecule at the allosteric site, as it was shown for CO and O_2_ escape rates and for cyanide binding when testosterone was present at the allosteric site [33,34].

## 5. Conclusions

In conclusion, we compared the rates of hydroxylation of two substrates PROG and MDZ by CYP3A4 in Nanodiscs assembled using various lipid compositions. Analysis of the NADPH oxidation rates and product forming rates revealed significantly different effects of lipid head-groups on the functional properties of CYP3A4 with these two substrates. While hydroxylation of PROG by CYP3A4 revealed significantly different distributions of product forming rates from the binding intermediates with one, two, and three substrates molecules bound to the CYP3A4 monomer, the results obtained for MDZ were only weakly affected by the difference in lipid composition. This difference is explained by the different affinity of the peripheral allosteric binding site formed between CYP3A4 F’ and G’ helices and lipid head-groups for PROG and for MDZ. In the case of PROG, the high affinity of this site when POPC is used for CYP3A4 assembly results in a lack of product from the first substrate binding event, and all products were generated from the fractions of CYP3A4 with two and three substrate molecules bound. When 40% DOPS and LPL were used for CYP3A4 incorporation in Nanodiscs, a significant rate of PROG hydroxylation was already observed with only one substrate molecule bound, and much higher rates were measured from the fraction with two substrate molecules. For MDZ variations in the lipid head-group, structure and charge were much less pronounced, mostly because of the low affinity of this substrate to the allosteric peripheral site. Overall, the results of this study demonstrate the importance of the affinity of this peripheral site of CYP3A4 in the membrane to a specific substrate, and highlight the significant contribution of interactions of P450 enzymes with the membrane to their functional properties.

## Figures and Tables

**Table 1:** Global fitting results for MDZ with two substrate binding sites and two products (1OH and 4OH hydroxy-MDZ). Stepwise dissociation constants K_d1_ and K_d2_, NADPH consumption rates v_1_ and v_2_, total product forming rates k_cat1_ and k_cat2_, and the fraction of the 4OH MDZ hydroxylation product from CYP3A4 monomer with one or two substrates bound were fitted simultaneously as described in Section 2. The coupling ratio is defined as the ratio of the NADPH consumption rate and product forming rate. The percentages of DOPE and DOPS are relative to a 100% POPC reference indicated.

	POPC	DOPE 40%	LPL	DOPS 40%
K_d1_, μM	3.6	3.4	3.4	1.8
K_d2_, μM	39.1	31.8	21.5	26.1
NADPH v_1_, min^−1^	119	183	171	194
NADPH v_2_, min^−1^	140	142	147	151
k_cat1_, min^−1^	5.1	6.0	6.0	4.3
k_cat2_, min^−1^	7.6	7.6	7.9	7.1
4OH fraction 1, %	5	4	1.5	1.8
4OH fraction 2, %	39	40	39	39
Coupling 1, %	4.3	3.3	3.5	2.2
Coupling 2, %	5.4	5.4	5.4	4.7

**Table 2:** Global fitting results for PROG with three substrate binding sites. Stepwise dissociation constants K_d1_, K_d2_, and K_d2_; NADPH consumption rates v_1_, v_2_, and v_3_; and total product forming rates k_cat1_, k_cat2_, and k_cat3_ from CYP3A4 monomer with one, two, or three substrates bound were fitted simultaneously, as described in Section 2. The coupling ratio is defined as the ratio of NADPH consumption rate and product forming rate.

	POPC	DOPE 40%	LPL	DOPS 40%
K_d1_, μM	15.5	20.3	16.9	26.4
K_d2_, μM	24.1	23.8	58.3	29.1
K_d3_, μM	26.8	26.5	64.8	34.3
NADPH v_1_, min^−1^	17	129	362	321
NADPH v_2_, min^−1^	550	437	334	303
NADPH v_3_, min^−1^	60	89	57	84
k_cat1_, min^−1^	0	0.4	10.3	16.9
k_cat2_, min^−1^	25.9	29.7	68.8	44.0
k_cat3_, min^−1^	18.3	20.5	11.6	25.1
Coupling 1, %	0	0.3	2.8	5.3
Coupling 2, %	4.7	6.8	20.6	14.5
Coupling 3, %	30.5	23.0	20.4	29.9

## Data Availability

Datasets are available on reasonable request from the authors.

## Supplementary Materials

The following supporting information can be downloaded at https://www.mdpi.com/article/10.3390/biology15020156/s1, Figure S1. UV-VIS spectra of substrate free CYP3A4 in Nanodiscs with different lipid composition; Figure S2. Global fit of turnover data with midazolam as a substrate in Nanodiscs with 100% POPC, calculated parameters are shown in Table 1; Figure S3. Global fit of turnover data with midazolam as a substrate in Nanodiscs with 40% DOPE, calculated parameters are shown in Table 1; Figure S4. Global fit of turnover data with midazolam as a substrate in Nanodiscs with liver polar lipids, calculated parameters are shown in Table 1; Figure S5. Global fit of turnover data with midazolam as a substrate in Nanodiscs with 40% DOPS, calculated parameters are shown in Table 1; Figure S6. NADPH oxidation rates divided by 5 (triangles) and progesterone hydroxylation rates (circles) by CYP3A4 in 100% POPC Nanodiscs. Results of a global fitting of the experimental data are shown together with calculated fractional contributions from CYP3A4 with one, two, or three substrate molecules bound; Figure S7. NADPH oxidation rates divided by 5 (triangles) and progesterone hydroxylation rates (circles) by CYP3A4 in 40% DOPE Nanodiscs. Results of a global fitting of experimental data are shown together with calculated fractional contributions from CYP3A4 with one, two, or three substrate molecules bound; Figure S8. NADPH oxidation rates divided by 5 (red triangles) and progesterone hydroxylation rates (blue circles) by CYP3A4 in 40% DOPS Nanodiscs. Results of a global fitting of experimental data are shown together with calculated fractional contributions from CYP3A4 with one, two, or three substrate molecules bound; Figure S9. NADPH oxidation rates divided by 5 (red triangles) and progesterone hydroxylation rates (blue circles) by CYP3A4 in liver polar lipids Nanodiscs. Results of a global fitting of experimental data are shown together with calculated fractional contributions from CYP3A4 with one, two, or three substrate molecules bound.
