# Supplementary material for: Lipid Dependence of CYP3A4 Activity in Nanodiscs
Source: Biology (Basel). 2026 Jan 15;15(2):156. doi: 10.3390/biology15020156 (PMC12837268; doi:10.3390/biology15020156)
Supplement: Supplementary file 1 [file biology-15-00156-s001.zip › biology-4069984-supplementary.pdf]

Supplemental Information figures

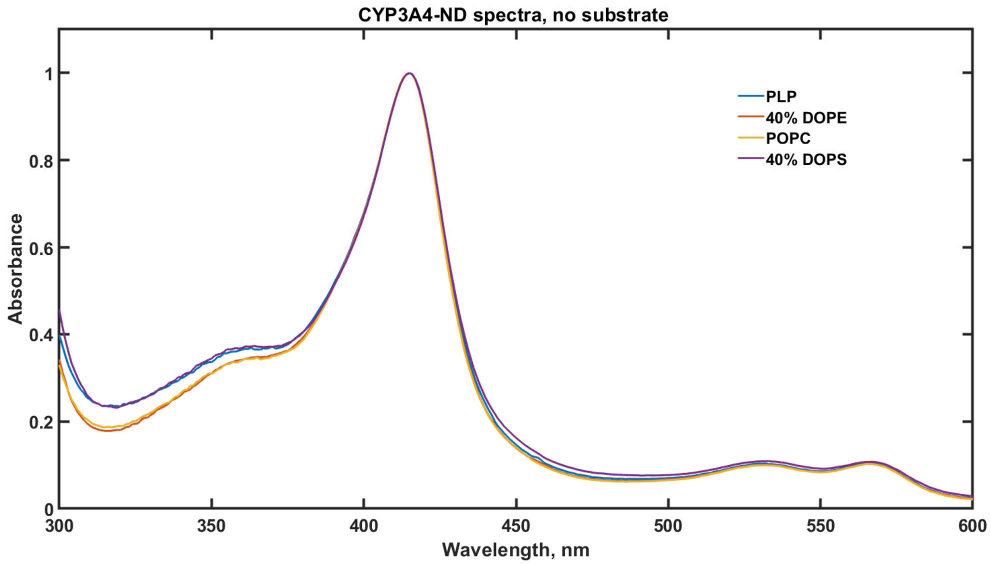

Figure S1. UV-VIS spectra of substrate free CYP3A4 in Nanodiscs with different lipid composition.

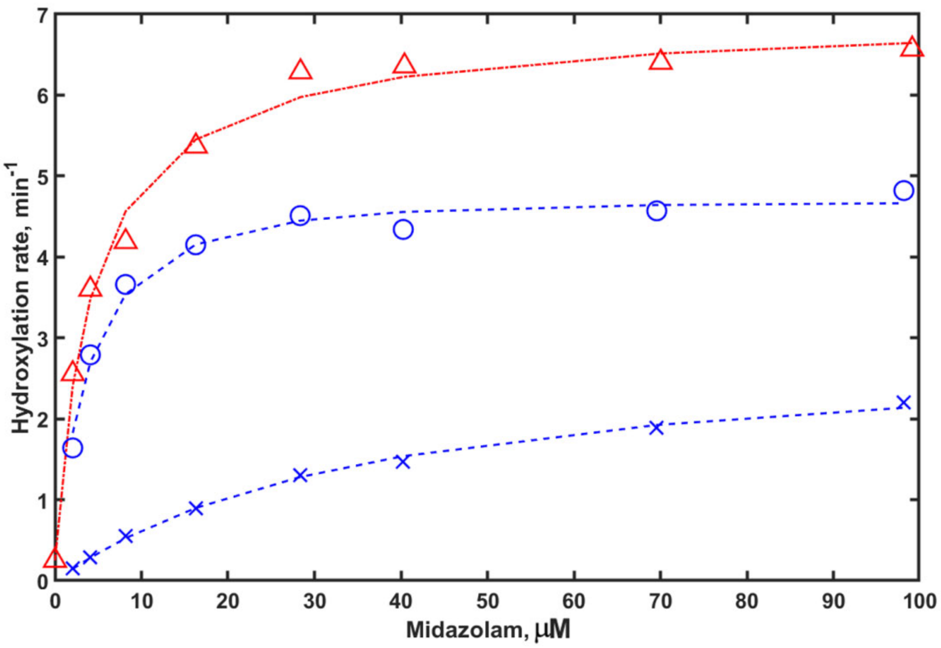

Figure S2. Global fit of turnover data with midazolam as a substrate in Nanodiscs with 100% POPC, calculated parameters are shown in Table 1

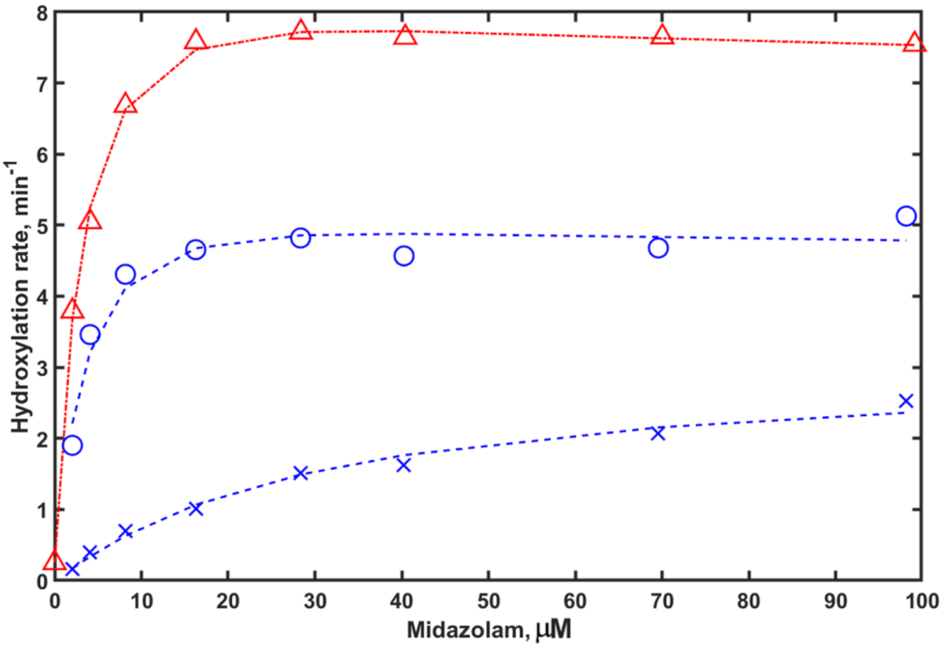

Figure S3. Global fit of turnover data with midazolam as a substrate in Nanodiscs with 40% DOPE, calculated parameters are shown in Table 1.

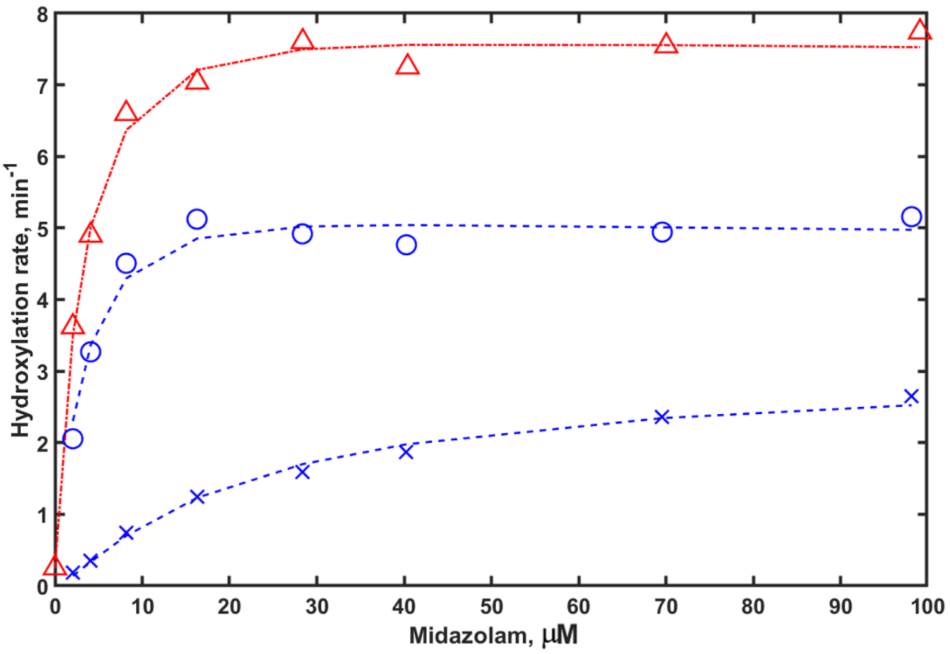

Figure S4. Global fit of turnover data with midazolam as a substrate in Nanodiscs with liver polar lipids, calculated parameters are shown in Table 1.

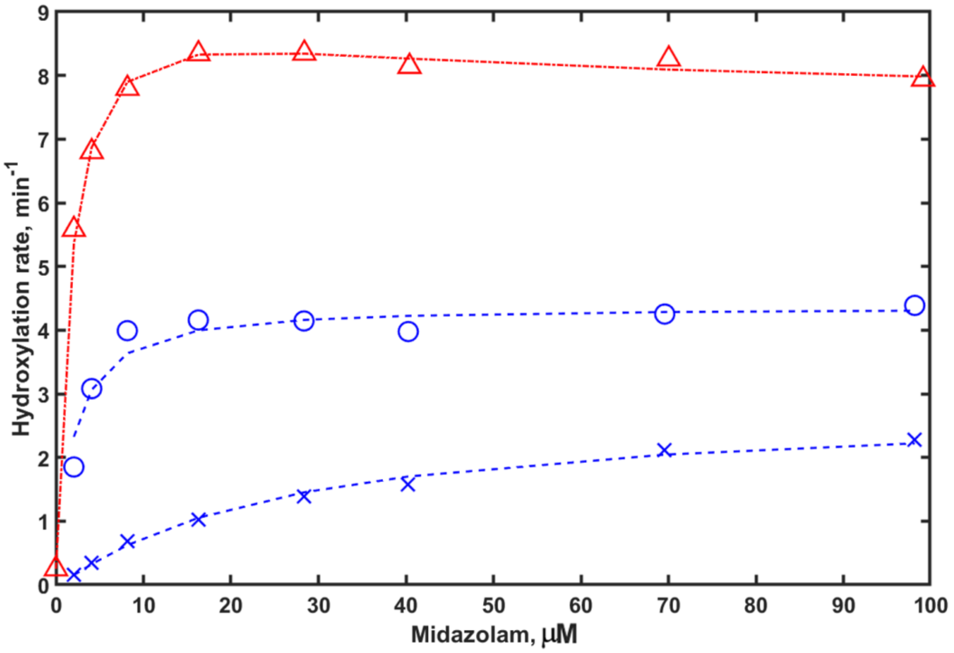

Figure S5. Global fit of turnover data with midazolam as a substrate in Nanodiscs with 40% DOPS, calculated parameters are shown in Table 1.

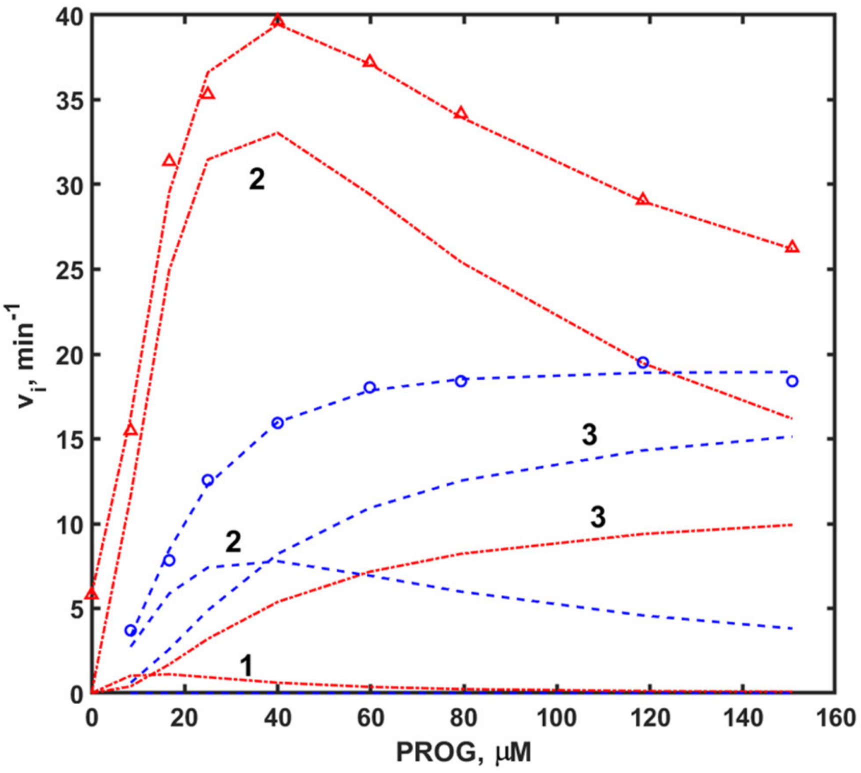

Figure S6. NADPH oxidation rates divided by 5 (triangles) and progesterone hydroxylation rates (circles) by CYP3A4 in 100% POPC Nanodiscs. Results of a global fitting of the experimental data are shown together with calculated fractional contributions from CYP3A4 with one, two, or three substrate molecules bound, indicated by corresponding numbers 1, 2, and 3 near the

curves. NADPH fitting results are shown in red dash-dot curves, progesterone hydroxylation rates are shown in blue dashed curves.

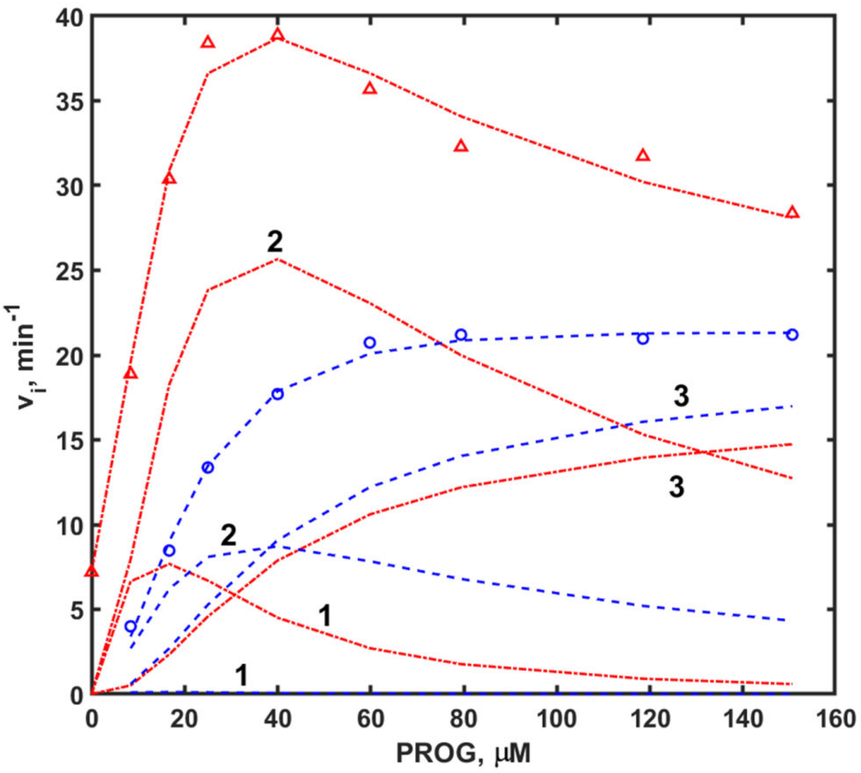

Figure S7. NADPH oxidation rates divided by 5 (triangles) and progesterone hydroxylation rates (circles) by CYP3A4 in 40% DOPE Nanodiscs. Results of a global fitting of experimental data are shown together with calculated fractional contributions from CYP3A4 with one, two, or three substrate molecules bound, indicated by corresponding numbers 1, 2, and 3 near the curves. NADPH fitting results are shown in red dash-dot curves, progesterone hydroxylation rates are shown in blue dashed curves.

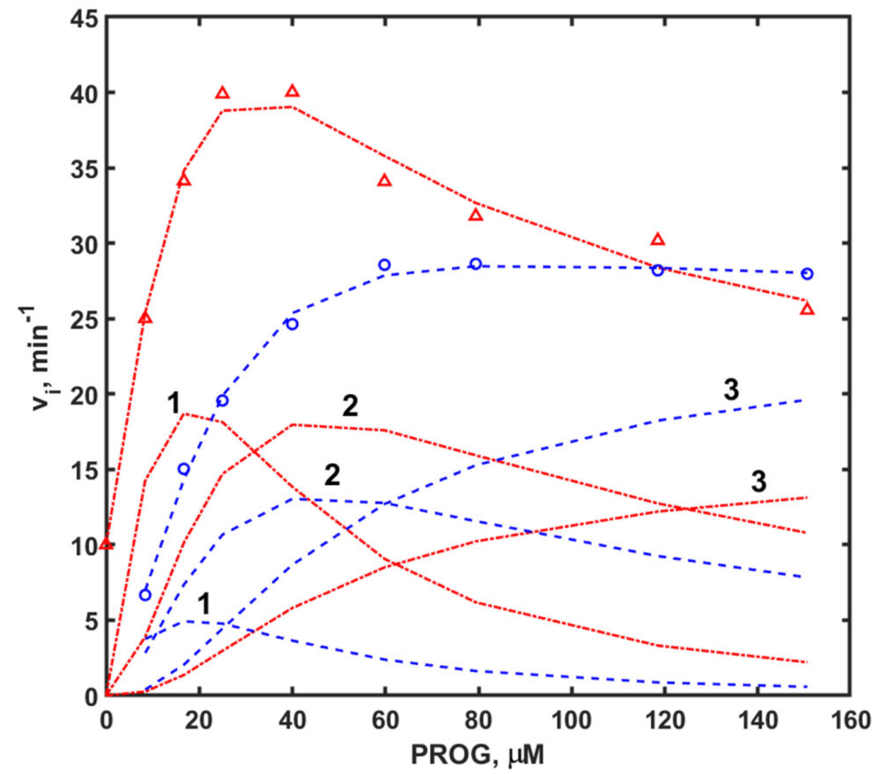

Figure S8. NADPH oxidation rates divided by 5 (red triangles) and progesterone hydroxylation rates (blue circles) by CYP3A4 in 40% DOPS Nanodiscs. Results of a global fitting of experimental data are shown together with calculated fractional contributions from CYP3A4 with one, two, or three substrate molecules bound, indicated by corresponding numbers 1, 2, and 3 near the curves. NADPH fitting results are shown in red dash-dot curves, progesterone hydroxylation rates are shown in blue dashed curves.

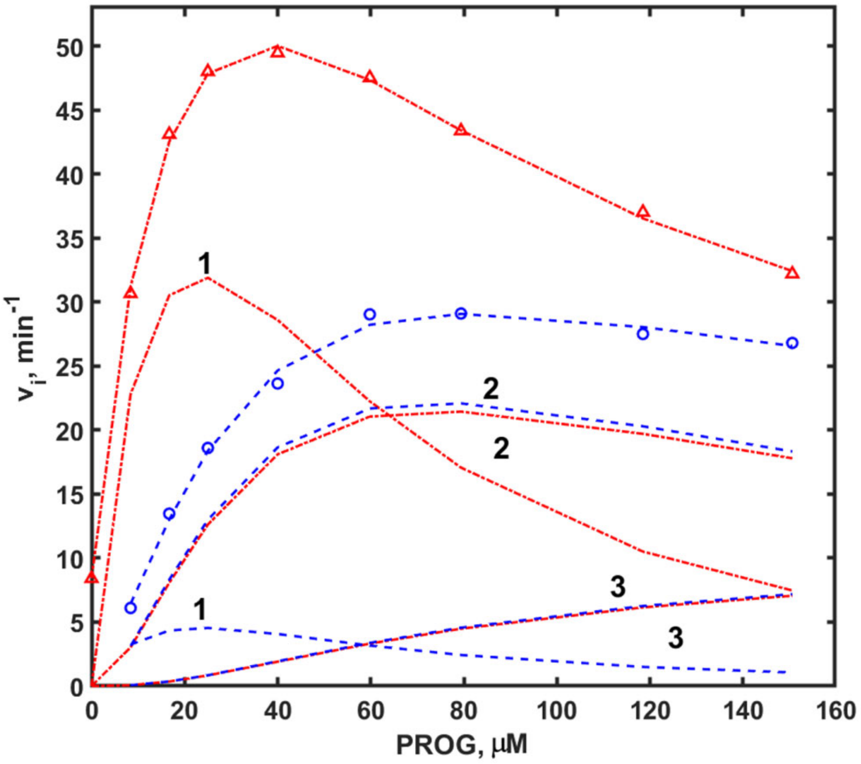

Figure S9. NADPH oxidation rates divided by 5 (red triangles) and progesterone hydroxylation rates (blue circles) by CYP3A4 in liver polar lipids Nanodiscs. Results of a global fitting of experimental data are shown together with calculated fractional contributions from CYP3A4 with one, two, or three substrate molecules bound, indicated by corresponding numbers 1, 2, and 3 near the curves. NADPH fitting results are shown in red dash-dot curves, progesterone hydroxylation rates are shown in blue dashed curves.
